# Supplementary material for: Site-Specific RNA Editing of Stop Mutations in the CFTR mRNA of Human Bronchial Cultured Cells
Source: Int J Mol Sci. 2023 Jun 30;24(13):10940. doi: 10.3390/ijms241310940 (PMC10342162; doi:10.3390/ijms241310940)
Supplement: Supplementary file 1 [file ijms-24-10940-s001.zip › ijms-2421367-supplementary.pdf]

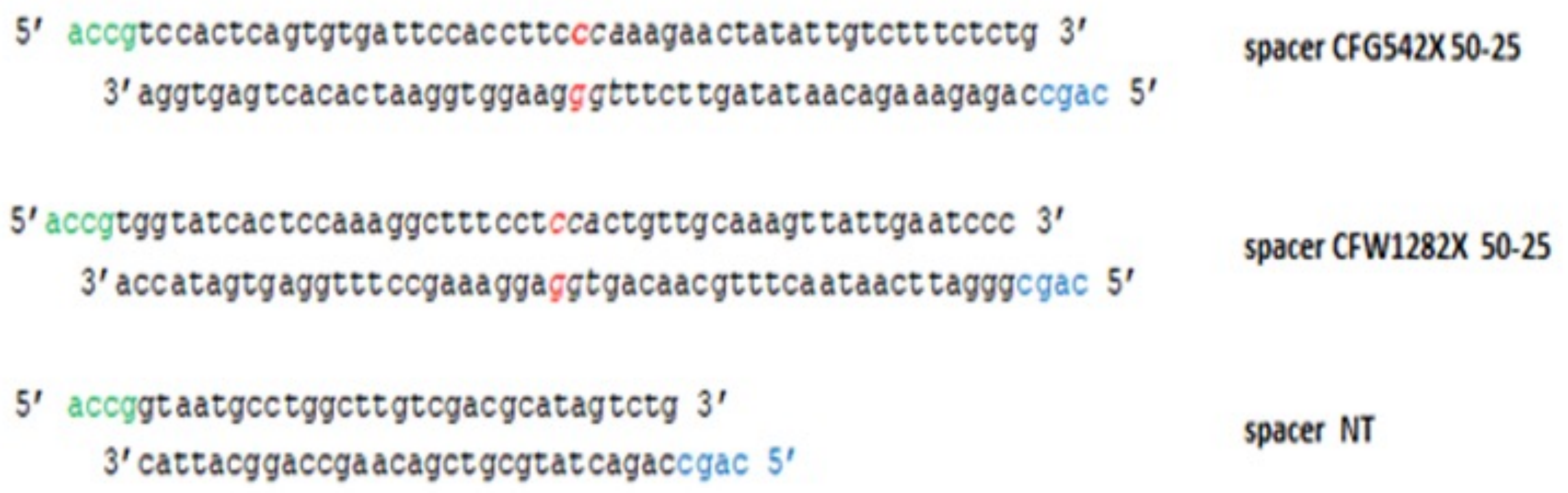

Figure S1. DNA Fragments cloned in the mxABE vector. Green or blue BbsI overhangs; red: mismatch position. The target mutation and the distance from the 5′ end of the sequence is specified in the name of each fragment. NT: Non-Targeting control fragment.

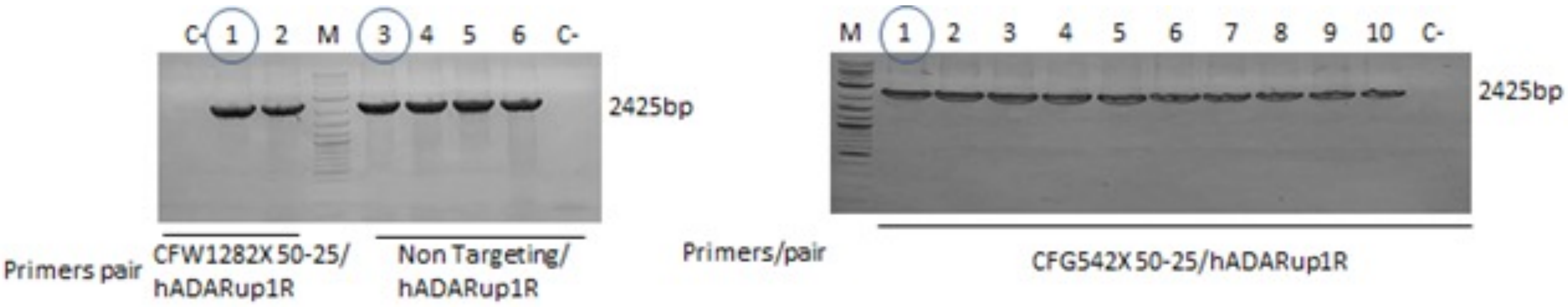

Figure S2. Colony PCR to select positive mxABE CFTR *g*RNA clones. Clone number, amplicon size and primers are shown. M: 2log ladder, Biolabs. Blue circles: selected clones.

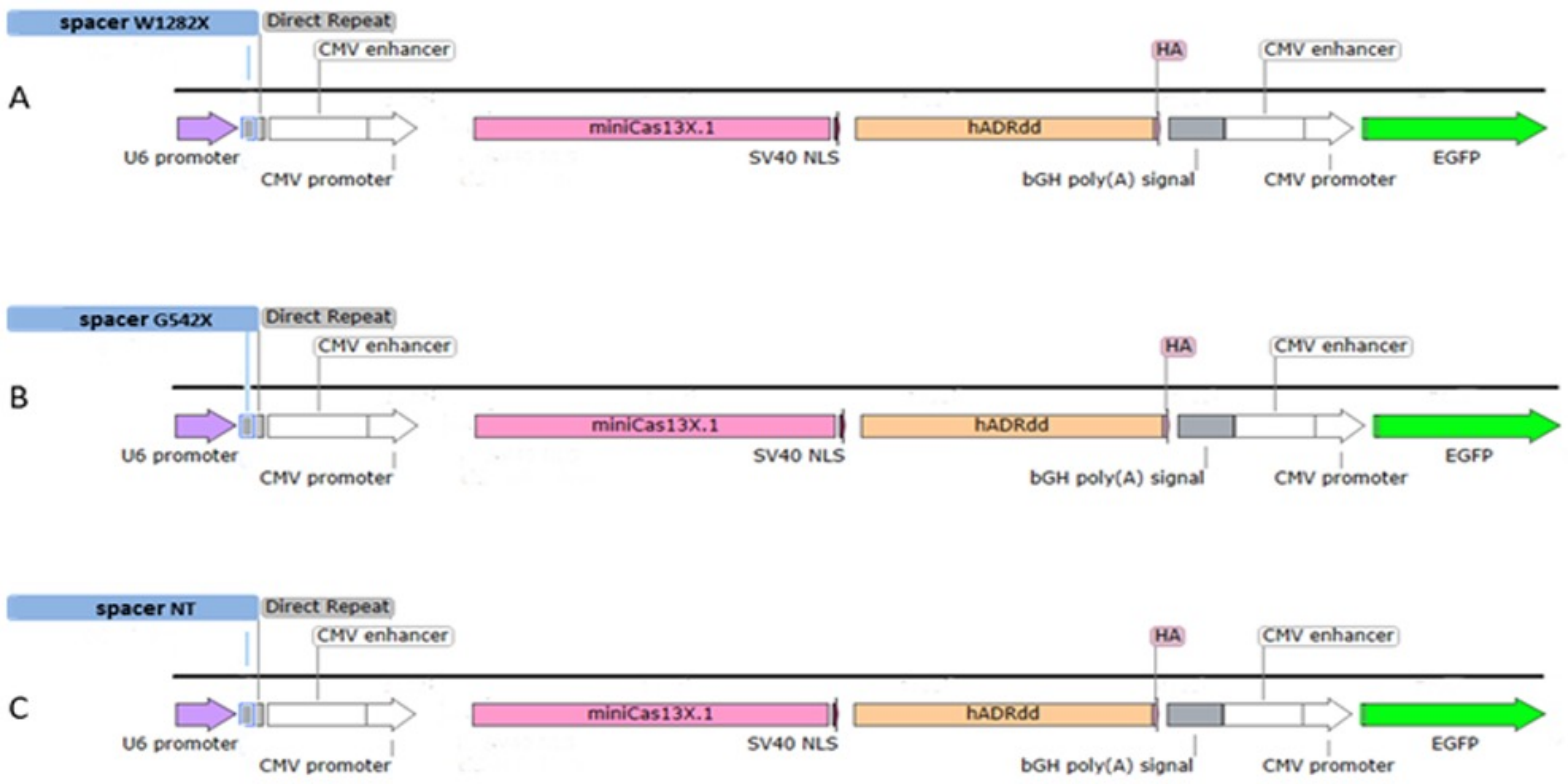

Figure S3. Linear map of the mxABE CFTR gRNA clones

Table S1: Oligonucleotides used in this study. F and dw: forward primers; R or up: reverse primers

Complementary oligonucleotides	Red bold: target position
G542X g13X.1 50-25 F	5’accgtccactcagtgtgattccaccttcccaaagaactatattgtctttctctg 3’
G542X g13X.1 50-25 R	5’cagccagagaaagacaatatagttctttgggaaggtggaatcacactgagtgga 3’
CFW1282X g13X.1 50-25 F	5’accgtggtatcactccaaaggctttcctccactgttgcaaagttattgaatccc
CFW1282X g13X.1 50-25R	5’cagcgggattcaataactttgcaacagtggaggaaagcctttggagtgatacca
Non Targeting g13X.1 F	5’accggtaatgcctggcttgtcgacgcatagtctg 3’
Non Targeting g13X.1 R	5’cagccagactatgcgtcgacaagccaggcattac 3’
Oligonucleotides used for RT-qPCR	
CFTR dw 13	5' atccctatgaacagtggag 3'
CFTR up 4	5' ttaggacacagccccccatc 3'
CFTR dw 10	5' acttctaatggtgatgacagcc 3'
CFTR up 9	5' atccagcaaccgccaacaact 3'
Oligonucleotide used for plasmid sequencing	
U6 forw	5' gagggcctatttcccatgattcc 3'

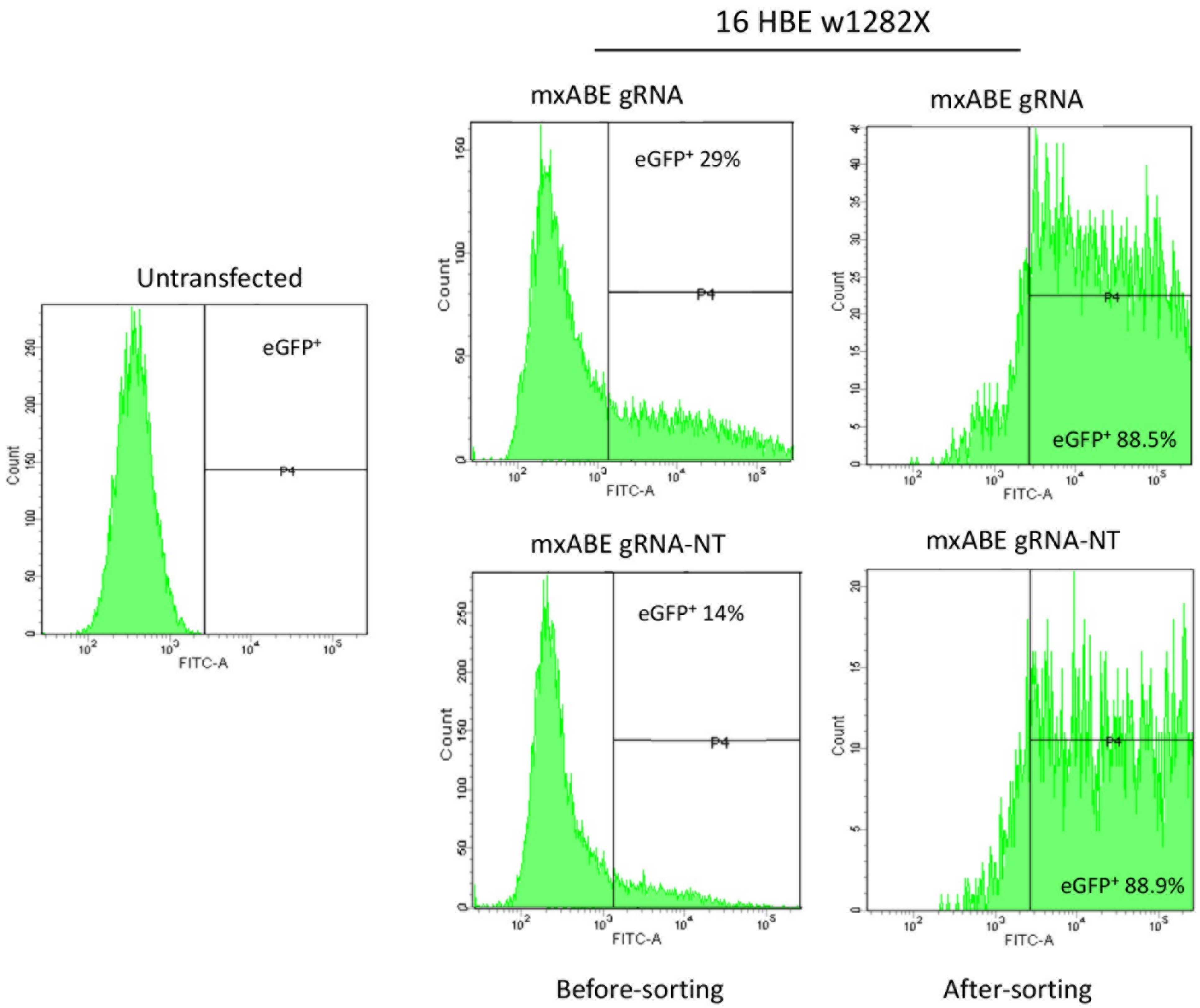

Figure S4. Cell sorting by cytofluorimetry. Untransfected and transfected cells were sorted for the presence of eGFP to enrich the cell population harboring the mxABE plasmid.

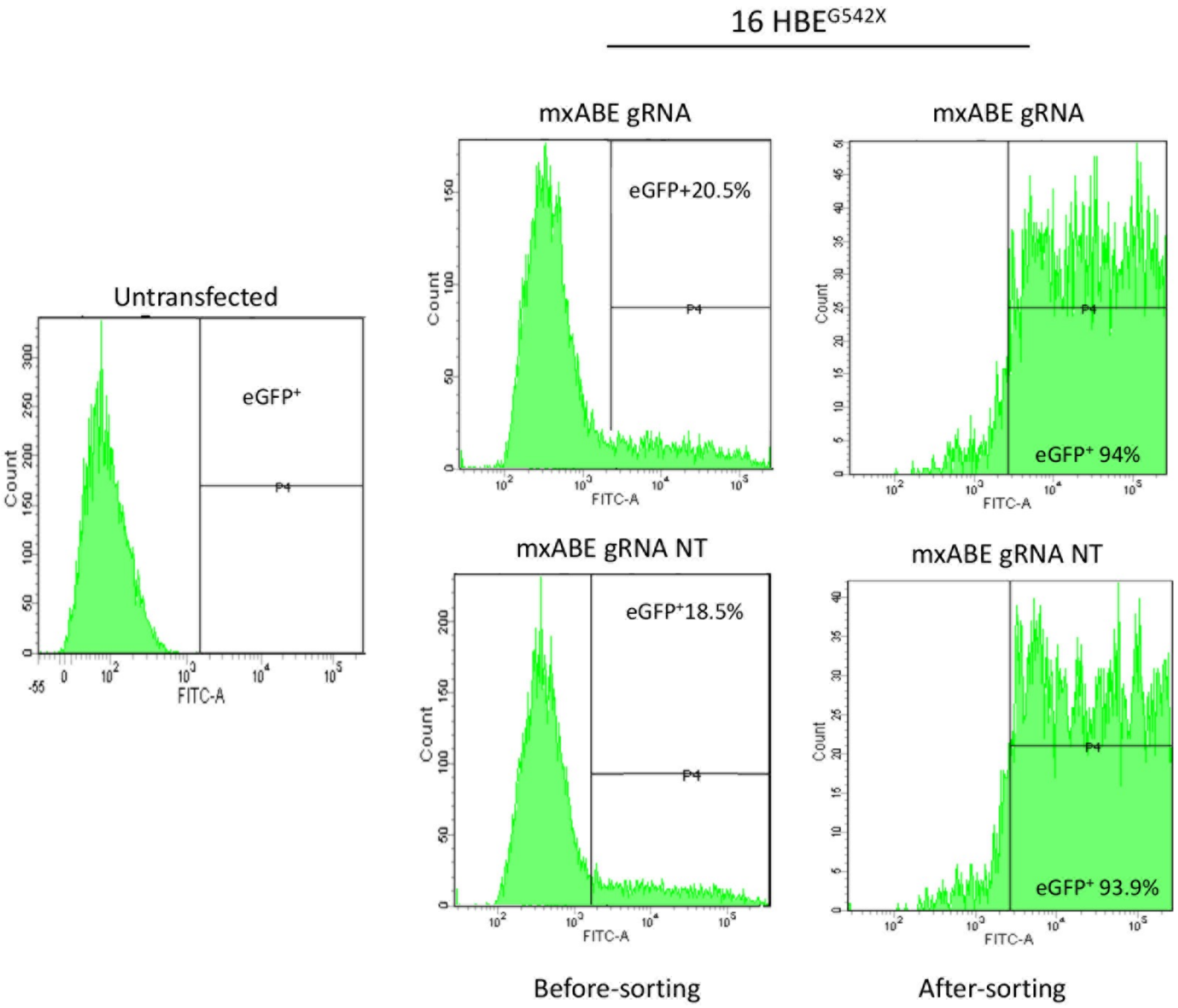

Figure S5. Cell sorting by cytofluorimetry. Untransfected and transfected cells were sorted for the presence of eGFP to enrich the cell population harboring the mxABE plasmid.

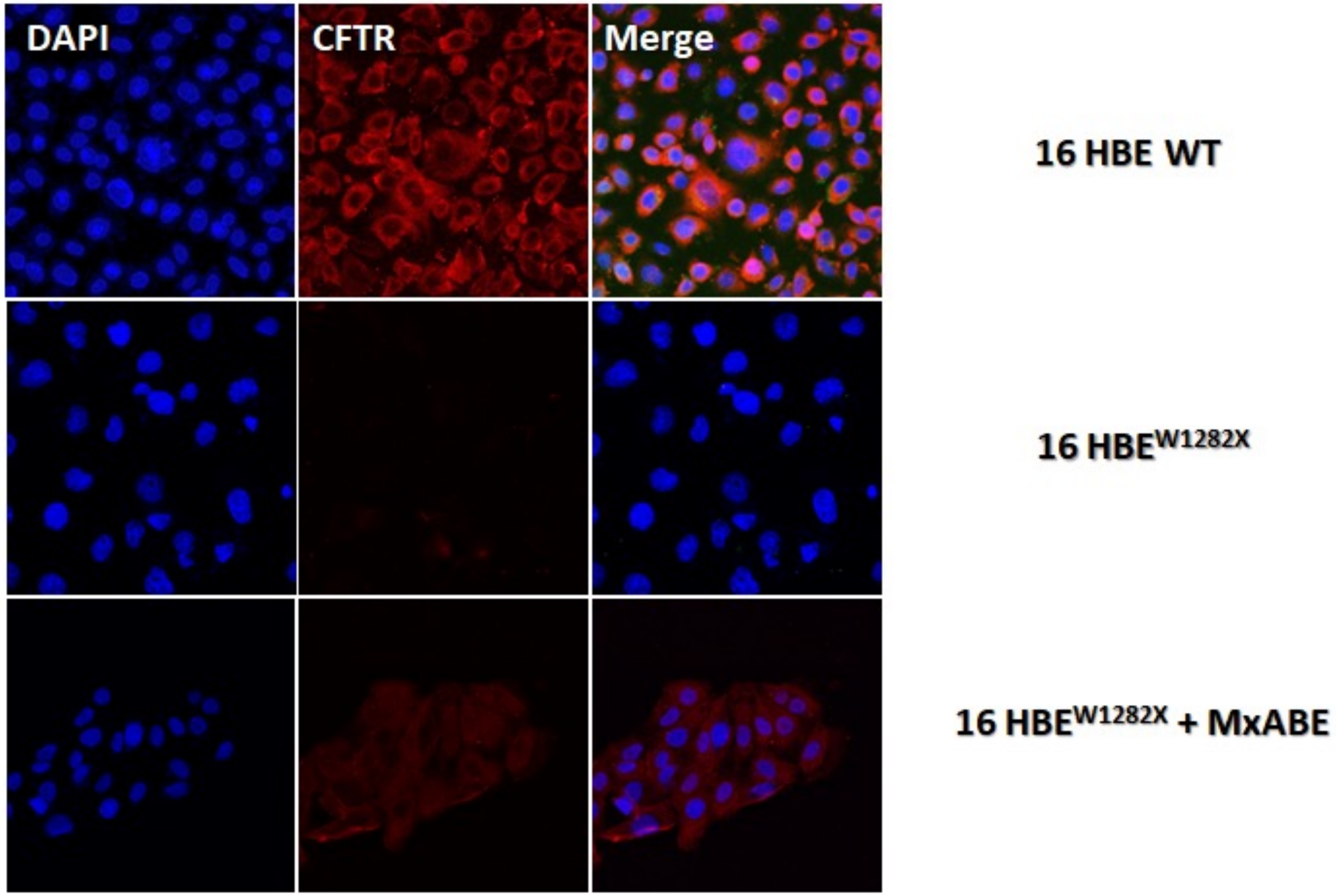

Figure S6. Confocal microscopy images of transfected cells.
